# Editorial: Molecular Mechanisms Controlling the Metabolic Reprogramming of Immune Cells Under Pathophysiological Conditions

**DOI:** 10.3389/fmolb.2020.00042

**Published:** 2020-03-06

**Authors:** Susan F. Fitzpatrick, Hannes Findeisen, Klaus Tenbrock

**Affiliations:** ^1^School of Medicine and Medical Science, Conway Institute, University College Dublin, Dublin, Ireland; ^2^Department of Cardiology i – Coronary and Peripheral Vascular Disease, Heart Failure, University Hospital Münster, Münster, Germany; ^3^Division of Pediatric Rheumatology, Department of Pediatrics, RWTH Aachen University, Aachen, Germany

**Keywords:** immunometabolism, immune cell, pathophysiology, molecular mechanisms, innate immunity, adaptive immune

Our innate (e.g., neutrophils, macrophages, monocytes, dendritic cells) and adaptive (e.g., T and B lymphocytes) immune cells have diverse functions, which are associated with distinct metabolic demands. Over the last decade we have witnessed the emergence of “immunometabolism” to describe the changes that occur in intracellular metabolic pathways during activation. While we now appreciate that profound metabolic changes occur in immune cells during normal physiological process and pathophysiology, more recent research has shed light on the molecular mechanisms governing these alterations. In time, we hope that this basic research can be translated into the development of novel therapies and diagnostic strategies for numerous diseases.

Here, we have collected review articles and abstracts, from some of the groups, focused on understanding the complex molecular mechanisms controlling the metabolic reprogramming of immune cells under pathophysiological conditions. We hope that this broad of discussion will provide a comprehensive overview of our current knowledge and open-up new avenues of research to explore.

Munford and Dimeloe provide a detailed review on intrinsic and extrinsic mechanisms of T cell metabolism in health and disease. They describe metabolic reprogramming during T cell activation and how this is linked to T cell function. They also describe intrinsic mechanisms of altered T cell function in different diseases like cancer including the role of PD1 as a checkpoint inhibitor target influencing metabolism of tumor cells as well as T cells. Regarding extrinsic factors that regulate T cell metabolism they put a focus on glucose availability, lactate, and other metabolites and the role of HIF1a in hypoxia.

Chambers and Matosevic review the role of hypoxia signaling on CD73 activity and how this effects natural killer (NK) cell metabolism in the tumor microenvironment. Specifically, they discuss the role of hypoxia inducible factor (HIF) the master regulator of the transcriptional response to hypoxia in controlling CD73, which in turn regulates glycolysis. In addition, CD73 also generates adenosine. Thus, the authors also examine adenosine signaling via the adenosine receptors, in particular Adenosine_2A_ receptor, in the dysfunction of NK cell metabolism. They conclude their discussion with a detailed overview on the potential of targeting the hypoxia-CD73 axis as an immunotherapeutic strategy for solid tumors.

Fitzpatrick initially provided a detailed overview of the changes which occur in the glycolytic, pentose phosphate, tricarboxylic acid cycle, and arginine metabolic pathways in innate immune cells in experimental models of sepsis and clinical sepsis. The second part of the review discusses the role of HIF in regulating these metabolic pathways during sepsis and the complex molecular mechanisms driving HIF expression. While the precise signaling mechanisms regulating HIF mediated metabolic changes remain to be fully elucidate, the author highlights that the situation is further complicated by the dynamics of the HIF response, the different roles of HIF in different immune cell populations and in response to different pathogens. In determining the validity of HIF as a therapeutic or diagnostic marker for sepsis these issues will need to be addressed.

Iron levels are rate limiting steps for bacterial growth and thus their regulation is of critical importance in host defense and immunity. Cronin et al. provided a very detailed review on iron metabolism at the cellular and organismal level starting at dietary and cellular uptake and mitochondrial utilization, furthermore describing intracellular storage and export from the cell. They then move on to immune-pathophysiological mechanisms of iron dysregulation and relevance of iron metabolism for different cell types like neutrophils, macrophages, and T cells. It finishes with tetrahydrobiopterin (BH4), which links T cell activation with iron metabolism in mitochondria. BH4 can reduce Fe^3+^ to Fe^2+^ and therefore affect cytochrome c mediated activity within the electron transport chain. The review concludes with an outlook of therapeutic interventions to target BH4 in diseases like cancer and autoimmunity.

Voss et al. present original research data on Fatty Acid Synthase that contributes to restimulation-induced cell death (RICD) of human CD4 T Cells. RICD is an apoptotic pathway triggered in activated effector T cells after T cell receptor engagement. They show that pharmacological blockade of fatty acid synthase significantly protected CD4 effector T cells from RICD. Decreased RICD in treated CD4 T cells correlated with reduced FAS ligand expression and a Th2-skewed phenotype, which appeared in previously naïve as well as memory T cells.

In response to *Mycobacterium tuberculosis* infection, host immune cells undergo considerable metabolic alterations. These immunometabolic adaptations are of significant importance, as they might decide the course of the infection. Kumar et al. offer us a detailed review of the involved immunometabolic networks regulating the host response to *Myocbacterium tuberculosis* infection. First, they focus on the metabolic reprogramming of specific phagocytic cell types such as macrophages, dendritic cells, and neutrophils. Then they provide a more detailed analysis of the involved molecular mechanisms with an in-depth description of important metabolic regulators, such as HIF1, mTOR, cMyc, PPARs, and others.

This collection of excellent articles covering various cell types, diseases, and physiologic pathways, highlights the fundamental importance of metabolic processes for immune cell function. Although there has been ground breaking progress in this field, we feel that we have only just begun to recognize the true significance. We are excited to participate in this research and are looking forward to any new discoveries.

## Author Contributions

All authors listed have made a substantial, direct and intellectual contribution to the work, and approved it for publication.

### Conflict of Interest

The authors declare that the research was conducted in the absence of any commercial or financial relationships that could be construed as a potential conflict of interest.

